# The “furrow sign” in confirming proper gastric extent and direction of the myotomy at the end of peroral endoscopic myotomy

**DOI:** 10.1055/a-2199-6956

**Published:** 2023-11-21

**Authors:** Hugo Uchima, Raquel Muñoz-González, Anna Calm, Noemí Caballero, Jorge Espinos, Vicente Moreno de Vega, Ingrid Marin

**Affiliations:** 1 Endoscopy Unit, Gastroenterology Department, Hospital Universitari Germans Trias i Pujol, Badalona, Spain; 2 Endoscopy Unit, Teknon Medical Center, Barcelona, Spain

Peroral endoscopic myotomy (POEM), first performed by Inoue in 2008, stands as a primary treatment for achalasia 1 . Ensuring the appropriate length of the gastric myotomy is crucial, as a myotomy that is too short can lead to an inadequate response. Conversely, excessive length can result in a higher incidence of moderate reflux esophagitis without enhancing clinical effectiveness 2 3 . While various landmarks and methods guide the proper extent of the tunnel 4 5 , a straightforward approach to confirm the end of the myotomy is lacking.

Herein, we introduce the concept of the “furrow sign,” characterized by a mucosal depression over a muscular defect in the wall post-myotomy. This sign becomes noticeable during gastric retroflexion under full insufflation, serving to confirm the extent and direction of the myotomy before closing the mucosal incision ( Fig. 1 ). To evaluate this sign, full carbon dioxide insufflation is applied in the gastric lumen for 60 seconds after myotomy ( Fig. 2 ).

**Fig. 1:**
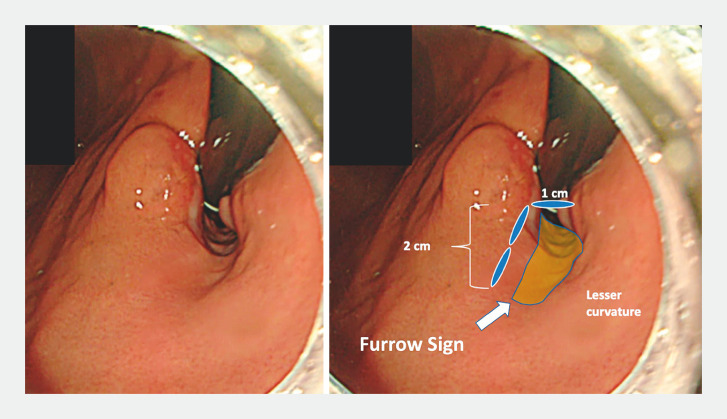
Endoscopic images of the “furrow sign,” characterized by a mucosal depression over a muscular defect in the wall post-myotomy after full carbon dioxide insufflation in the stomach.

**Fig. 2:**
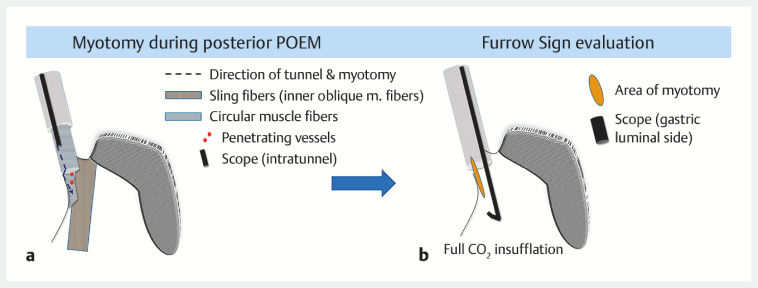
Evaluation of the “furrow sign”. **a** After myotomy. **b** “Furrow sign” is evaluated by applying full carbon dioxide insufflation in the gastric lumen for 60 seconds.

Between May and September 2022, we assessed this sign in seven patients who underwent posterior POEM with comprehensive follow-up. Clinical success was evident in all cases ( Table 1 ), and no adverse events linked to full carbon dioxide insufflation during furrow sign evaluation were recorded. On each occasion, the furrow sign was confirmed by at least two endoscopists.

**Table:** **Table 1** Characteristics and follow-up of patients in whom the “furrow sign” was evaluated during posterior peroral endoscopic myotomy (May–September 2022).

Baseline and follow-up characteristics	N=7
Baseline	
Age, median (IQR), years	63 (54–72)
Female sex, n (%)	5 (71.4)
Type of achalasia, n (%)	
Type II	5 (71.4)
Type III	2 (28.6)
Pre-POEM IRP, median (IQR)	27.3 (20.2–30.4)
Pre-POEM Eckardt score, median (IQR)	7 (6–7)
Follow-up, median (IQR)	
Gastric myotomy length, cm	2 (2–2.5)
Post-POEM Eckardt score	0 (0–0)
Supine IRP post-POEM, mmHg	13.3 (11.4–15)
Upright IRP post-POEM, mmHg	8.1 (5.5–9.6)
Time until HRM, months	4 (3–5)
IQR, interquartile range; POEM, peroral endoscopic myotomy; IRP, integrated relaxation pressure; HRM, high resolution manometry.	

We verified via double-scope transillumination that the depression area seen in the furrow sign aligns with the myotomy area ( Fig. 3 ), with its persistence observed even 12 months after POEM ( Video 1 ).

**Fig. 3:**
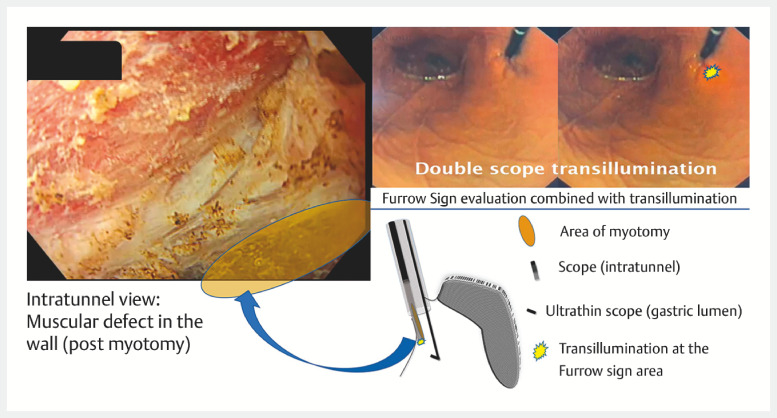
The depression area seen in the furrow sign aligns with the myotomy area, as confirmed by double-scope transillumination.

Video clinical cases that demonstrate the practical application and utility of the “furrow sign.”Video 1

In conclusion, the furrow sign corresponds to the myotomy area and can play a crucial role in confirming or fine tuning the accurate length (and also confirming the direction) of the gastric myotomy at the conclusion of the POEM procedure, prior to closing the mucosal incision. To establish its prevalence, sensitivity, specificity, and interobserver agreement, further studies are imperative.

Endoscopy_UCTN_Code_TTT_1AO_2AN

## Notes

Endoscopy E-Videos https://eref.thieme.de/e-videos
*E-Videos* is an open access online section of the journal *Endoscopy* , reporting on interesting cases and new techniques in gastroenterological endoscopy. All papers include a high-quality video and are published with a Creative Commons CC-BY license. Endoscopy E-Videos qualify for HINARI discounts and waivers and eligibility is automatically checked during the submission process. We grant 100% waivers to articles whose corresponding authors are based in Group A countries and 50% waivers to those who are based in Group B countries as classified by Research4Life (see: https://www.research4life.org/access/eligibility/ ). This section has its own submission website at https://mc.manuscriptcentral.com/e-videos .
